# Anatomical variations of the celiac trunk and hepatic arterial system: an analysis using multidetector computed tomography angiography*

**DOI:** 10.1590/0100-3984.2014.0100

**Published:** 2015

**Authors:** Severino Aires Araujo Neto, Henrique Almeida Franca, Carlos Fernando de Mello Júnior, Eulâmpio José Silva Neto, Gustavo Ramalho Pessoa Negromonte, Cláudia Martina Araújo Duarte, Bartolomeu Fragoso Cavalcanti Neto, Rebeca Danielly da Fonseca Farias

**Affiliations:** 1 PhD, Associate Professor II of Medical Radiology, Universidade Federal da Paraíba (UFPB), João Pessoa, PB, Brazil.; 2 Graduate Students of Medicine at Universidade Federal da Paraíba (UFPB), João Pessoa, PB, Brazil.; 3 PhD, Associate Professor IV of Medical Radiology, Universidade Federal da Paraíba (UFPB), João Pessoa, PB, Brazil.; 4 PhD, Associate Professor II of Anatomy, Universidade Federal da Paraíba (UFPB), João Pessoa, PB, Brazil.

**Keywords:** Anatomy, Celiac trunk, Hepatic artery, Multidetector computed tomography

## Abstract

**Objective:**

To analyze the prevalence of anatomical variations of celiac arterial trunk (CAT) branches and hepatic arterial system (HAS), as well as the CAT diameter, length and distance to the superior mesenteric artery.

**Materials and Methods:**

Retrospective, cross-sectional and predominantly descriptive study based on the analysis of multidetector computed tomography images of 60 patients.

**Results:**

The celiac trunk anatomy was normal in 90% of cases. Hepatosplenic trunk was found in 8.3% of patients, and hepatogastric trunk in 1.7%. Variation of the HAS was observed in 21.7% of cases, including anomalous location of the right hepatic artery in 8.3% of cases, and of the left hepatic artery, in 5%. Also, cases of joint relocation of right and left hepatic arteries, and trifurcation of the proper hepatic artery were observed, respectively, in 3 (5%) and 2 (3.3%) patients. Mean length and caliber of the CAT were 2.3 cm and 0.8 cm, respectively. Mean distance between CAT and superior mesenteric artery was 1.2 cm (standard deviation = 4.08). A significant correlation was observed between CAT diameter and length, and CAT diameter and distance to superior mesenteric artery.

**Conclusion:**

The pattern of CAT variations and diameter corroborate the majority of the literature data. However, this does not happen in relation to the HAS.

## Abstract (pt)

**Objetivo:**

Analisar a prevalência de variações anatômicas da ramificação do tronco arterial celíaco (TAC) e do sistema arterial hepático (SAH), o diâmetro e comprimento do TAC e sua distância para a artéria mesentérica superior.

**Materiais e Métodos:**

Estudo retrospectivo, transversal, predominantemente descritivo, baseado na análise de imagens de tomografia computadorizada de 60 pacientes.

**Resultados:**

A anatomia do TAC foi normal em 90% dos casos. Cinco (8,3%) pacientes apresentaram o tronco hepatoesplênico e um (1,7%) apresentou o tronco hepatogástrico. O SAH variou em 21,7% dos casos. Desses, 8,3% foram na localização anômala da artéria hepática direita e 5% da artéria hepática esquerda. Ainda foram encontrados 3 (5%) casos de relocalização conjunta da artéria hepática direita e artéria hepática esquerda e 2 (3,3%) de trifurcação da artéria hepática própria. A média de comprimento e o calibre médio do TAC foram, respectivamente, 2,33 cm e 0,8 cm. A distância média entre o TAC e a artéria mesentérica superior foi 1,2 cm, com desviopadrão de 4,08. Houve correlação significativa entre diâmetro e comprimento do TAC, e diâmetro do TAC e distância deste para a artéria mesentérica superior.

**Conclusão:**

O padrão de variação do TAC e seu diâmetro corroboram a maioria dos dados da literatura, embora o mesmo não tenha ocorrido em relação ao SAH.

## INTRODUCTION

Recent developments in surgical techniques, in organ transplants and in imaging methods help physicians to make decisions regarding the most appropriate choice of the several therapeutic possibilities, either surgical and non-surgical. However, many times, physicians face anatomical variations that can impair diagnosis or the performance of surgical procedures. Thus, it is important to have appropriate knowledge on the human anatomy and its most frequent variations that affect a population^(1-3)^.

With the introduction of minimally invasive surgeries, catheterization and abdominal angioplasty, the study of the celiac trunk diameter and length, as well as its distance to the upper mesenteric artery became necessary for a better preoperative planning.

The celiac arterial trunk (CAT) emerges immediately after the aortic hiatus, at the level of the T12 thoracic vertebra, and its normal pattern is related to the origin of three branches, namely, the left gastric artery, which runs along the lesser curvature of the stomach; the splenic artery, which tortuously runs to the spleen; and the common hepatic artery, which breaks into gastroduodenal artery, in the vascularization of the pancreas and duodenum, and the hepatic artery proper which supplies the liver. The trifurcation is reported in the literature as occurring in 89% of the cases, while bifurcation occurs in 11%. Absence of this trunk occurs in 0.2% of the individuals^(4)^. However, other authors report a greater number of variations. Mburu et al. report to have found the trifurcation pattern in only 61.7% of 123 dissected bodies^(5)^.

The hepatic arterial system (HAS) described as normal is characterized as a right and left hepatic artery coming from the hepatic artery proper, which, by its turn, originates from the common hepatic artery, after emergence of the gastroduodenal artery, which runs inferiorly. The division of the hepatic artery proper into right and left hepatic arteries should occur proximally to the liver within the hepatoduodenal ligament.

According to Ugurel et al.^(6)^, in a retrospective study of 100 computed tomography (CT) images, HAS with variations was found in 48% of the cases. Sebben et al., in a study of 30 cases, have reported variation in 40% of their cases^(7)^, while the Sobotta Anatomy Atlas records variation of this artery in 35% of the cases^(8)^. Iezzi et al.^(9)^, on its turn, has found a variation of 27.9% in a sample of 524 cases.

The length of the CAT varied when a Greek study was compared with a Brazilian one, however, the diameter values were very similar^(10)^. Considering their results, Silveira et al.^(11)^ have suggested that further studies should be undertaken in order to confirm the variation in CAT length and diameter.

Such a wide diversity of data extracted from different populations exposes the fragility of scientific evidence in this topic, leading to the questioning of the universal and indiscriminate applicability of one or another reference source in the medical practice, particularly when populations with different ethnicities are considered. Thus, the development of studies with Brazilian populations on the anatomical pattern of such arteries is desirable and pertinent.

The present study is aimed at analyzing the prevalence of anatomical variations of CAT and HAS ramifications, utilizing images from patients submitted to abdominal intravenous contrast-enhanced multidetector CT scans.

## MATERIALS AND METHODS

The images utilized in the present review were retrieved from the authors' personal files, and were acquired with a 64-channel multidetector tomography apparatus, model Brilliance (Philips Medical System; The Netherlands). The present study was duly approved by the Committee for Ethics in Research of Universidade Federal da Paraíba.

The scan protocol, with small sporadic variations, consisted of axial 1 mm-thick slices, with a pitch of 0.8. The utilized contrast agent was Ultravist (Bayer), in the concentration of 769 mg/mL, intravenously injected by an injection pump, at a rate of 5 mL/s, employing bolus tracking with fixed time delay and field-of-view of 250 mm (standard). The reconstruction thickness of the images was 2 mm and was performed on an Extended Brilliance Work Space workstation with the software Philips Brilliance for tomography.

In order to define the arterial pattern, analyses were carried out in the axial plane, reconstruction techniques in the coronal and sagittal planes on multiplanar reconstructions (MPR), as well as three-dimensional (3D) reconstructions with the maximum intensity projection (MIP) and volume rendering (VTR) techniques. The normal pattern and the main variations of CAT and HAS were demonstrated.

The study was retrospective, cross-sectional, predominantly descriptive, based on the analysis of multidetector CT images from outpatients at a private clinic in the city of João Pessoa, PB, Brazil.

The study sample comprised all CT studies of the abdominal region (either upper or total abdomen) performed with iodinated contrast medium injection, for various indications, including CT angiography of the region. The following items were evaluated: a) pattern of ramification and distribution of the celiac trunk; b) pattern of ramification and distribution of the HAS; c) length of the celiac trunk; d) celiac trunk caliber; e) distance between the emergence of the celiac trunk and the superior mesenteric artery.

The analysis of the images was carried out by an experienced radiologist. The study sample included 60 cases, and the analysis covered all the above described items.

Patients presenting with aneurysm or abdominal aortic prosthesis, history of liver or gastric surgery and/or poor image quality including those caused by failure in intravenous contrast uptake in the studied vessels, were not included in the analysis.

For the analysis of the obtained data, a statistical method was selected, based on the adherence to the model of normal distribution and equality of variance. The value of *p* < 0.05 was considered as being statistically significant. The Statistical Package for Social Sciences (SPSS) 19.0 was utilized for statistics calculations. For the coefficient of variation and correlation analyses, the Spearman, Pearson and Yates methods included in the SPSS were utilized.

## RESULTS

**Figure 1:**
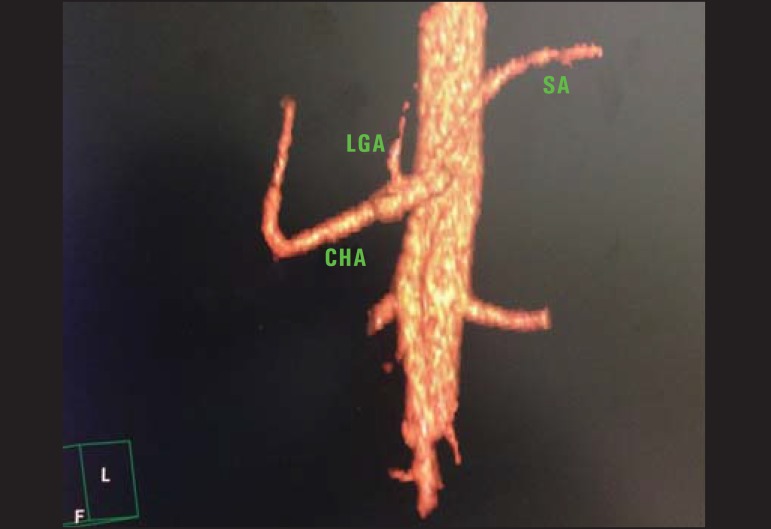
Contrast-enhanced CT with volume rendering shows normal anatomy of the celiac trunk comprising the left gastric artery (LGA), splenic artery (SA) and common hepatic artery (CHA).

**Figure 2:**
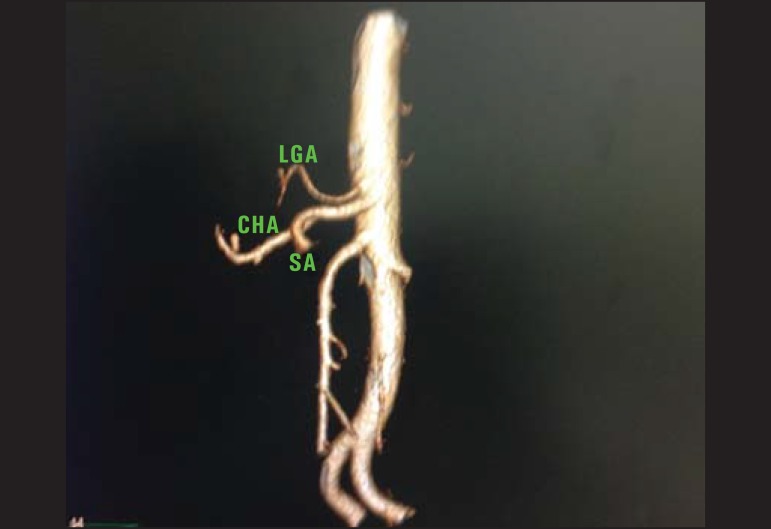
Contrast-enhanced CT with volume rendering demonstrating hepatosplenic trunk with left gastric artery (LGA) originating in the aorta, immediately above the trunk. The splenic artery (SA) and common hepatic artery (CHA) originate from the same trunk.

Out of the 60 cases included in the present study, 35 were women and 25 were men. An n value corresponding to 54 (94%) was classified as normal CAT anatomy (trifurcation into left gastric artery, splenic artery and common hepatic artery) (Figure 1). In the five cases of hepatosplenic trunk (8.3%) (Figure 2) the left gastric artery originated from the aorta. In the case of hepatogastric trunk, the splenic artery also emerged from the aorta (1.7%).

**Figure 3:**
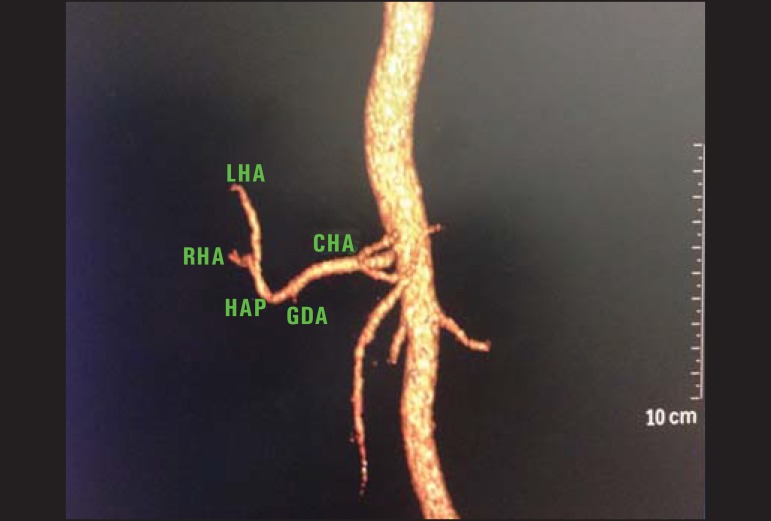
Contrast-enhanced CT with volume rendering showing normal anatomy of the hepatic arterial system. Common hepatic artery (CHA) originating the hepatic artery proper (HAP), after emergence of the gastroduodenal artery (GDA), right hepatic artery (RHA) and left hepatic artery (LHA) originating from HAP.

**Figure 4:**
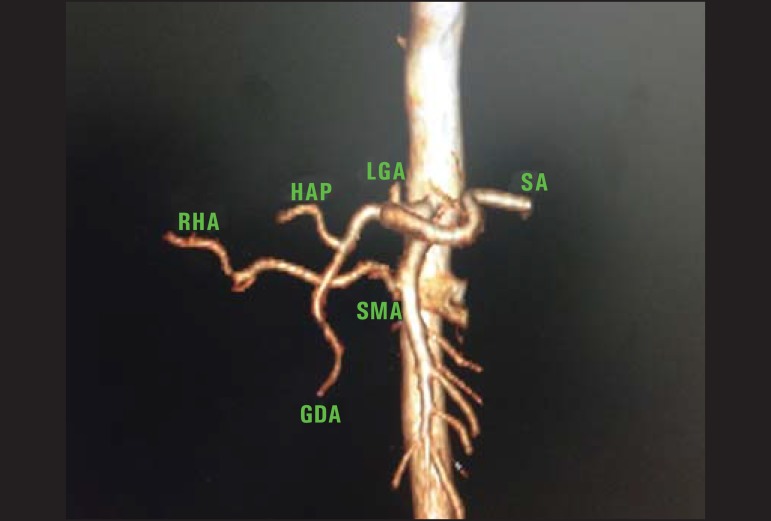
Contrast-enhanced CT with volume rendering identifying variation of the right hepatic artery (RHA) that emerges from the superior mesenteric artery (SMA). (LGA, left gastric artery; SA, splenic artery; HAP, hepatic artery proper; GDA, gastroduodenal artery).

The HAS varied in 13 cases (21.7%), with 78.3% of the cases presenting with normal anatomy (Figure 3). Anomalous right hepatic artery location was the most frequently found anatomical variation, in 5 cases (8.5%), in three of them originating from the mesenteric artery (5.1%) (Figure 4) and, in two cases, from the CAT (3.4%). In the three cases of left hepatic artery abnormalities (5.1%), two emerged from the common hepatic artery (3.4%) and one from the left gastric artery (1.7%). Three cases showed joint relocation of the left and right hepatic arteries (5.1%). The two cases of trifurcation of the hepatic artery proper presented with a middle hepatic artery.

**Figure 5:**
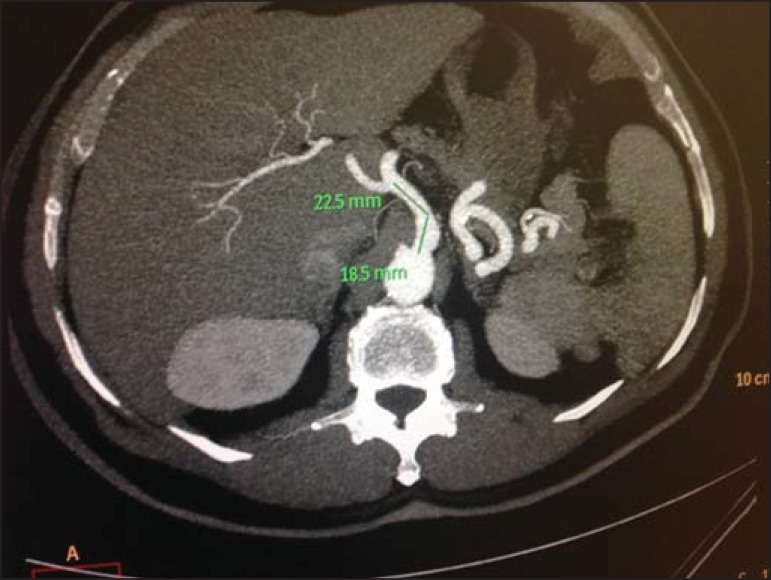
Axial, contrast-enhanced CT showing the longer length of the celiac trunk observed in the present study.

**Figure 6:**
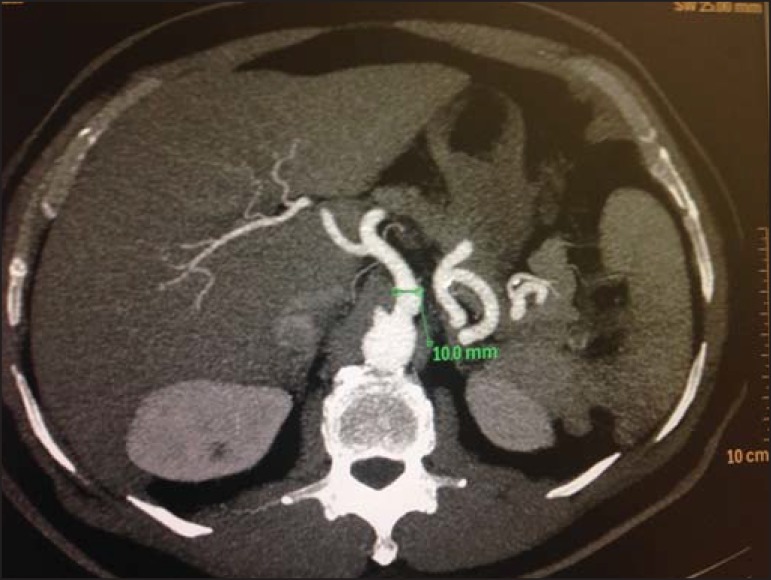
Axial contrast-enhanced CT showing the largest diameter observed in the present study.

On average, the length of CAT was 2.33 cm, the longest with 4.1 cm (Figure 5) and the shortest, 1.0 cm, and standard deviation of 0.65. On average, the CAT caliber was 0.8 cm, the largest with 1 cm (Figure 6) and the smallest, 0.5 cm, and standard deviation of 0.13. The mean distance between the CAT and the superior mesenteric artery was 1.2 cm, the largest being 2.3 cm, and the shortest, 0.3 cm, with standard deviation of 0.4.

No correlation was observed between CAT length and its distance to the superior mesenteric artery (*p* = 0.068). Pearson's correlation was utilized for such calculations. A positive correlation with moderate magnitude was observed between CAT length and diameter (*p* = 0.001; *n* = 60; ρ = 0.43), showing that increased celiac trunk length is associated with increased diameter. Spearman's correlation was utilized for such calculations. A positive correlation with low magnitude was observed CAT diameter and distance to the superior mesenteric artery (*p* = 0.049; *n* = 60; ρ = 0.255), showing that increased diameter is correlated with increased distance between those two arteries. Spearman's correlation was utilized for such calculations.

## DISCUSSION

In the present study, the variation in CAT ramification confirmed data reported by most authors, who describe the finding of normality in approximately 90% of cases, as found in most studies^(12-14)^ (Table 1).

**Table 1:** Normal pattern and variation of the celiac trunk in percentage.

Study	Country	*N* of the study	Method	Normal pattern	Variation
Current study	Brazil	60	Multidetector CT	90%	10%
Iezzi et al.^(9)^	Italy	555	Multidetector CT	72.1%	27.9%
Koops et al.^(18)^	Germany	604	Multidetector CT	79.1%	20.9%
Panagouli et al.^(10)^	Greece, Caucasians	62	Cadaver dissection	88.5%	11.5%
Prakash et al.^(3)^	India	50	Cadaver dissection	86%	14%
Silveira et al.^(11)^	Brazil	21	Cadaver dissection	71.4%	28.6%
Song et al.^(13)^	South Korea	5002	Multidetector CT	89.1%	10.1%
Sureka et al.^(14)^	India	600	Multidetector CT	94.5%	5.5%
Ugurel et al.^(6)^	Turkey	100	Multidetector CT	89%	11%

The HAS variation was smaller than that reported by Ugurel et al.^(6)^ (*n* = 100 cases; variation: 48%), Sebben et al.^(7)^ (*n* = 40 cases; variation: 40%), and the Sobotta Anatomy Atlas^(8)^ that records variations in 35% of the individuals. The studies developed by Pérez-Saborido et al.^(15)^ (*n* = 325), Gümüs et al.^(16)^ (*n* = 820), Chambers et al.^(17)^ (*n* = 50), Koops et al.^(18)^ (*n* = 604), and mainly the Brazilian studies developed by Freitas et al.^(19)^ (*n* = 246) and Bertevello et al.^(20)^ (*n* = 60) present results similar to the ones found in our investigation, with HAS variation in 27.9% of cases (Table 2).

**Table 2:** Normal pattern and HAS variation.

Study	Country	*N* of the study	Method	Normal pattern	Variation
Perez-Saborido et al.^(15)^	Spain	325	Surgical	78%	22%
Gumus et al.^(16)^	Turkey	820	Multidetector CT	76.8%	23.2%
Sebben et al.^(7)^	Brazil	30	Cadaver dissection	60%	40%
Freitas et al.^(19)^	Brazil	246	Surgical	76.8%%	23.2%
Bertevello et al.^(20)^	Brazil	60	Cadaver dissection	68.3%	31.7%
Chambers et al.^(17)^	United States of America	50	Multidetector CT	84%	16%
Koops et al.^(18)^	Germany	604	Multidetector CT	79.1%	20.9%
Sureka et al.^(14)^	India	600	Multidetector CT	79.6%	20.4%
Song et al.^(13)^	South Korea	5002	Multidetector CT	58%	42%
Ugurel et al.^(6)^	Turkey	100	Multidetector CT	52%	48%

The study developed in cadavers by Panagouli et al.^(10)^ defined the mean CAT length as 2.6 cm, with a difference of 0.3 cm in relation to the present study, where the mean length was 2.3 cm. The CAT caliber was practically identical to that reported by Silveira et al.^(11)^, of 0.79 cm, in a study on cadavers. Millimetric differences in CAT length and diameter may be justified by different forms of measurement. In the study developed by Panagouli et al.^(10)^ and Silveira et al.^(11)^ the data were collected by means of rulers on cadavers, while in the present investigation such measurement was obtained by means of a software specific for radiology.

As regards distance from the superior mesenteric artery to the CAT, the present study found a mean value of 1.2 cm, with a difference of 0.7 cm less than the value found by Panagouli et al.^(10)^ in dissections. Some of the justifications that may be pointed out for such a considerable difference of 24% include the different ethnicities, as the study by Panagouli et al. was developed in Greece, with Caucasians, and the differences in methods employed in the two studies.

The moderate positive correlation observed between CAT diameter and its length (*p* = 0.001) was not found in other studies and neither was the mild positive correlation (*p* = 0.049) found between the TAC diameter and its distance to the superior mesenteric artery.

The mentioned correlations are fundamental for the surgeon to know the exact site of the origin of the arteries, as well as the length of such vessels and the interval in which such arteries may originate. Such data are important not only in vascular surgeries, but also to avoid iatrogenesis in any surgery. The data from the present study also are useful in laparoscopic and robotic surgery, considering the limited operative field in such surgical modalities. The knowledge of the diameter and length of the vessels also influence on surgeries for placement of arterial stents, as it is also useful for professionals who design and develop the stents^(10)^.

Another important feature of such measurements lies on the fact that X-ray angiography techniques are routinely utilized, but do not allow for the visualization of the vessels. In this context, surgeons many times choose the vessel for infusion of the contrast medium by trial and error based on their own personal experience, which offers a great probability of errors. Therefore, considering that abdominal angiography takes vertebral bodies as a reference, it is indispensable to know the position of the vessels originating from the aorta, such as the celiac trunk and respective branches and the superior mesenteric artery, as well as the distance between them^(21)^.

When choosing the size of the catheter, it is also fundamental to know the diameter of the vessels in order to prevent iatrogenic injuries to such vessels^(11)^.

The knowledge of the CAT diameter is useful in the radiological diagnosis of arterial stenosis. The literature presents several studies approaching the celiac trunk and its branches, however few studies approach the diameter of such structures^(11)^.
